# Employing multiple synchronous outcome samples per subject to improve study efficiency

**DOI:** 10.1186/s12874-021-01414-7

**Published:** 2021-10-17

**Authors:** Roger P. A’Hern

**Affiliations:** London, UK

**Keywords:** Clinical trials, Sample size, Multiple synchronous samples, Cluster design

## Abstract

**Background:**

Accuracy can be improved by taking multiple synchronous samples from each subject in a study to estimate the endpoint of interest if sample values are not highly correlated. If feasible, it is useful to assess the value of this cluster approach when planning studies. Multiple assessments may be the only method to increase power to an acceptable level if the number of subjects is limited.

**Methods:**

The main aim is to estimate the difference in outcome between groups of subjects by taking one or more synchronous primary outcome samples or measurements. A summary statistic from multiple samples per subject will typically have a lower sampling error. The number of subjects can be balanced against the number of synchronous samples to minimize the sampling error, subject to design constraints. This approach can include estimating the optimum number of samples given the cost per subject and the cost per sample.

**Results:**

The accuracy improvement achieved by taking multiple samples depends on the intra-class correlation (ICC). The lower the ICC, the greater the benefit that can accrue. If the ICC is high, then a second sample will provide little additional information about the subject’s true value. If the ICC is very low, adding a sample can be equivalent to adding an extra subject. Benefits of multiple samples include the ability to reduce the number of subjects in a study and increase both the power and the available alpha. If, for example, the ICC is 35%, adding a second measurement can be equivalent to adding 48% more subjects to a single measurement study.

**Conclusion:**

A study’s design can sometimes be improved by taking multiple synchronous samples. It is useful to evaluate this strategy as an extension of a single sample design. An Excel workbook is provided to allow researchers to explore the most appropriate number of samples to take in a given setting.

**Supplementary Information:**

The online version contains supplementary material available at 10.1186/s12874-021-01414-7.

## Background

In some circumstances, it is possible to undertake more than one synchronous assessment of the same subject to estimate a measure of interest. The overall result might then be calculated as the average across the assessments or perhaps as the maximum or minimum value if they are more critical. It is natural to ask whether it is worth making multiple measurements to improve the assessment quality. This has to be weighed against any disadvantages - an assessment may be burdensome to the subject or clinician/scientist undertaking it or more costly. This paper offers a quantitative framework, including easy-to-use software, for making this decision, developed from first principles to outline its basis. The overall variance is the sum of the between-subject and within-subject variance, so reducing the effect of within-subject variability by performing repeat within-subject observations can be beneficial. As might be anticipated, this strategy is most useful when the within-subject variation is a large proportion of the overall variation, equivalent to a low to moderate intraclass correlation (ICC) between observations.

Improving study design by taking repeated measurements may be particularly valuable if the number of subjects eligible for the study is limited, for example, in rare disease types or restricted subject groups. The degree of correlation between repeated measurements is important. For example, if the ICC were 85%, taking more than one sample would offer little additional information about the subject’s actual value.

If the correlation is 10% or lower, multiple samples might be considered if there were reasonable grounds for this degree of independence. However, in other circumstances, it may be concluded the parameter being measured was of little value because of low repeatability.

As a practical example, many human organs are bilateral, and if a sample were taken from each, these would show correlated results because of their shared genotypic and environmental background. Is it worth taking measurements from both organs to study organ function? The eye provides a useful illustration. Glynn and Rosner [1] presented alternative models for predicting the percent of normal visual field in 197 subjects being followed up for glaucoma. Inclusion of both eyes (*N* = 394) in a mixed-effects analysis, which allows for correlation, was found to improve the accuracy of the estimates of potential predictive factors compared with the use of only one eye. The correlation between the eyes was 0.55 (55%). A median reduction in the standard error of the parameter estimates in the 394 ‘eye’ based analysis relative to the single eye analysis was 15% (for the factor hypertension) with a range of 12% (for gender) to 39% (for acuity loss). Reductions of this order are worthwhile, a 15% reduction in the standard error could, for example, increase the power to detect a real difference from 66 to 80%, and a 39% reduction could increase the power from 40 to 80%.

From another viewpoint, employing a summary value from several measurements rather than the value of a single measure can be seen as improving the ICC by lowering the within-subject variation. For example, Lee et al. [2] studied the ICC’s of 3 representative physical examinations - popliteal angle, Thomas test, and Staheli test, performed twice each by three orthopedic surgeons on 30 cerebral palsy subjects with a mean age of 12.5 years. The popliteal angle test is a measure of hamstring tightness; both the Thomas test and the Staheli test are methods of measuring hip flexion contracture. The single test ICC’s were 0.71, 0.46, and 0.22. However, the ICC’s of the averages of the three assessments were 0.88, 0.74, and 0.46, respectively, suggesting the popliteal test can be improved and a more satisfactory ICC for the Thomas test can be achieved if they are applied independently multiple times.

Examples of specific contexts in which this technique might be used will now be considered. A sample size calculation based on the above Glynn and Rosner example suggests that a standardized difference of 0.4 in a binary predictive factor could be detected with 80% power using 197 single eyes; this decreases to a standardized difference of 0.25 if both eyes (*N* = 394) are studied. Nicholson and Holmes [3] noted that a popular but improper method for assessing high-throughput assays’ precision is by scatter-plotting data. This consists of equally dividing a sample and assaying the two halves separately, then plotting and correlating all analytes’ results in the first half versus the second half. They concluded that precision should not be based on all analytes’ plots. The repeatability of individual analytes and a variance inflation factor should be used to calculate appropriate sample sizes to detect changes in specific analyte levels that are the focus of the researcher’s interest. The biased scatter-plotting method typically gives ‘excellent’ correlations of 0.95 or greater, but for four high throughput assays Nicholson and Holmes reported ICCs of 0.31 (0.10-0.53) [Median (IQ range)] for 1624 microRNA analytes, 0.59 (0.24-0.80) for 17,788 mRNA analytes, 0.31 (0.20-0.50) for 69 proteins and 0.94 (0.82-0.96) for 163 metabolites. This suggests ICCs for some analytes in high-throughput assays are at levels that may make repeat samples worthwhile. Ionan et al. [4] described the National Cancer Institute’s Director’s Challenge reproducibility study results. This examined the reproducibility of 22,283 features from the Affymetrix U133A Genechip across a collection of 11 frozen patient tissue samples. These were assayed at four different labs. Fifty-percent of the 22,283 ICC’s were below 0.52, and 25% were below 0.23.

The majority of ICC values derived from adjustment factors calculated in a study of UK Biobank data by Morgan et al. [5] were observed to be above 70%, but not all. The following are below 70%: Diastolic blood pressure (60% (95%CI:60-62)); Systolic blood pressure (65% (95%CI: 64-65)); Pulse rate (62% (95%CI:61-64)); Peak expiratory flow (60%(95%CI:59-61)) and Grip strength (65% (95%CI:63-67)). All hematological factors in this study and a meta-analysis by Coskuna et al. [6] had ICCs that were 70% or greater. Multiple measurements are used to measure physical activity. For example, Lee et al. [7] found 3 days accelerometer testing was adequate in South Korean patients with stroke receiving inpatient rehabilitation but noted that conventionally 7 days testing was used [8]. It is also recommended that blood pressure is measured over several different days and while resting to get reliable readings [9]. More complex situations can arise if multiple measures with differing numbers of measures per subject are used to calculate an endpoint. In studies of advanced cancer, in which subjects may have cancer present at multiple sites, the within-participant sum of tumor lesion diameters is used for overall tumor response calculation in RECIST [10]. This sum will decrease if there is a positive response to therapy. Caution was initially exercised; before 2009, the recommendation was to measure up to 10 lesions per subject but subsequently [11], a maximum of 5 was found to be sufficient. This supports the existence of correlation of the responses of different lesions from the same subject.

Random measurement error occurs when measurements vary unpredictably around their true values and is the result of both true biological variability and imprecise measurement techniques. It presents a widespread challenge in clinical practice and medical research [12]. This paper considers random error in the outcome being assessed but random error can also play an important role in independent variables that are used to predict outcome, or independent variables used as stratification factors. For example, if the relationship of left ventricular mass to blood pressure was being assessed the random error in the measurement of both left ventricular mass and blood pressure would be crucial. In this case an increase in the error in the measurement of blood pressure would cause a weakening of its predicted relationship to left ventricular volume. Hutcheson, Chiolero and Hanley [12] provide an excellent guide to this phenomenon. Correction factors can be applied to address this issue, if they are known. For example, relevant correction factors from the UK Biobank have been provided by Morgan et al. [5]. If the effect of a randomised treatment intervention on left ventricular mass was being assessed the random error in mass measurement would be important, but typically there would be no error associated with the allocation of treatment groups, but confounding could occur if subjects did not adhere to their randomised treatment.

The potential value of synchronous measurements is also supported by recognizing that slope (linear trend) estimation can be optimized by making as many observations as feasible at the extreme ends of the ranges of independent variables. For example, to estimate a linear relationship (decrease or increase) within a subject over 2 years by making six measurements, three measures at baseline and three at 24 m would yield a more accurate estimate of change than spacing the measurements, such as one each at baseline, 4 m, 8 m, 12 m, 18 m and 24 m. However, caution needs to be exercised because trends may not be linear and confounders can be important. For example, Aadland et al. [13] noted that systematic reviews had generally concluded that there was no association between physical activity and BMI in children less than 7 years old. However, in their study of 1120 Norwegian children, these authors found that physical activity was more clearly negatively associated with BMI in older children in this age group and in girls compared to boys.

In this paper, subjects are considered the basic experimental unit of interest, and single or multiple assessments, measurements, or samples are taken from these subjects. The methodology is not new but is identical to that of cluster randomized trials. However, the focus is on samples within subjects rather than subjects within clusters. The software presented can also be used for cluster designs by considering the cluster units as subjects. For simplicity, the main focus is on situations where it is possible to average measures across repeated samples or for binary endpoints, the probability that each measure takes one of the true possible values, e.g. that it is a ‘success’.

## Implementation

The variance of the mean of several identically normally distributed random variables can be calculated by noting that for two variables, the variance Var(aX+bY) = a^2^Var(X) + b^2^Var(Y) + 2abCovar(X,Y). If X and Y have mean (X + Y)/2, ρ is their correlation and Var(X) = Var(Y) = s^2^, then Covar(X,Y) is ρs^2^ and a = b = 1/2. Hence Var(Mean) = s^2^/4 + s2/4 + 2ρs^2^/4 = (s^2^/2)(1 + ρ). By extension, it can be shown that the variance of the mean of m samples is Var(Mean) = (s^2^/m)(1 + (m-1)ρ). In the absence of correlation, the variance would be s^2^/m; hence the quantity (m-1)ρ is the variance increase due to the correlation, and (1 + (m-1)ρ) is known as the Variance Inflation Factor (VIF). If m is not consistent across the subjects, it is typically replaced by the mean value across subjects; however, the coefficient of variation (CV) of m can also be incorporated to estimate the variance (see below). These formulae mirror those used in cluster randomized trials, programs performing sample size calculation for cluster randomized trials can therefore be used in the current context.

**Fig. 1:**
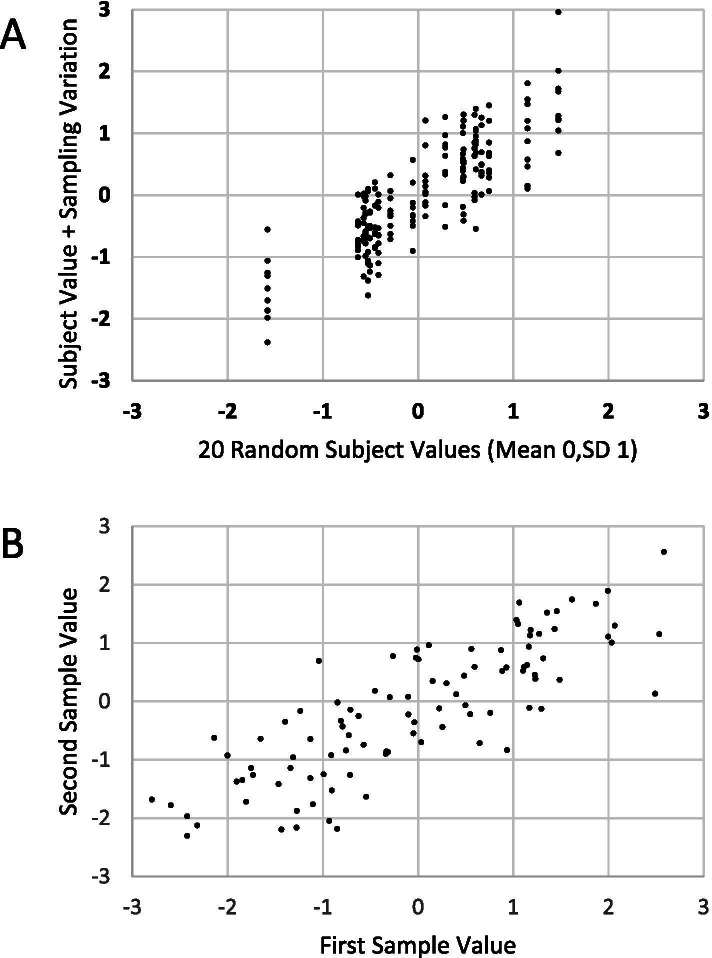
**A** and **B** Illustrate the joint effects of between and within subject variation and their contribution to correlation. Please see text for more detail

A fundamental relationship, shown in Fig. 1A and B, is worth noting. If subjects have normally distributed average values with variance V_subj_, and samples have values normally distributed about these averages with variance V_samp_, then the intraclass correlation (ICC or ρ) is V_subj_/(V_subj_ + V_samp_). Figure 1A shows this diagrammatically for a randomly simulated sample with V_subj_ = 1 and V_samp_ = 0.25, an ICCof 0.8. In this simulated data, the subject means have been defined to vary randomly about zero, and there are 20 subjects each with ten assessments. This correlation is also apparent when plotting within-subject observations, plotting pairs of values from the same subject from this simulated data (Fig. 1B). The correlation in this context will typically be close to but not the same as the ICC.

An Excel Workbook (STARS.xlsx) is provided, which includes worksheets to illustrate sample size calculation incorporating the number of measures, the ICC, and the CV of the number of measures, including details on the optimum ‘cost’ based choice of the number of samples. Sample size worksheets for estimating ICC are also included, as are examples demonstrating techniques of analysis and associated commands in the programming language R.

STARS has been designed to calculate sample sizes for two group comparisons, hence it cannot be used for studies testing effect size of a single group, such as might be the case in a single group Phase II trial. It also cannot be used for studies examining change in a single group, e.g. those which intend to use a paired t-test or McNemar’s test for analysis. It is pertinent to consider longitudinal and other complex designs, two sources of correlation need to be considered. In the case of longitudinal designs, these are the correlation between measures at the same timepoint and the correlation between measures at different timepoints.

The simplest longitudinal designs are those with paired scores, e.g. baseline (pre-test) and post-test. The within subject correlation is also important in this case and in many situations the focus can be the single ‘change’ endpoint (post-pre), and its standard deviation will be known from previous experience. If the intention is to have the same number of measures pre and post and the ICC at both these timepoints is the same, then the standard deviation and ICC can be used in STARS to estimate the optimal number of measures. The example below uses this method.

If the standard deviation of the change is not known, assuming pre and post scores have the same SD (s), and the correlation τ between them is known, then the change can be shown to have an SD of √(2s^2^ - 2τs^2^). In this situation, the design contrast **D** can be represented by **D** = (− 1, 1)^1^ and the variance covariance matrix as **V**, equal to

$$\left.\left(\begin{array}{c}{s}^2\ {\tau s}^2\\ {}{\tau s}^2\ {s}^2\end{array}\right.\right)$$

The SD is then √(**DVD**^T^) = √(2σ^2^ - 2τσ^2^), where **D**^T^ is **D** transposed. Generally, for any design contrast **D** and any variance covariance matrix **V**, multiple serial scores per subject can be combined into a single endpoint **DX**^T^ with SD given by √(**DVD**^T^). It is therefore relevant to consider how an appropriate variance/covariance matrix can be identified to allow this to be done from a specimen dataset, which represents the structure of the data contributing to the study to be undertaken. STARS illustrates how this can be accomplished using R for four scenarios (‘Correlation between Repeated Measures’ option). However, STARS may not always be suitable for sample size calculation in this context and simulation may be more relevant.

### Sample size considerations

For continuous normal and binomial comparisons standard sample size formulae have been employed. For example, to detect a difference of D then it is typically desirable that the standard error (SE) of the estimated difference is approximately D/3 (the ‘Rule of Three’). This is on the basis that if a two-sided 5% significance level is employed then this will require the estimated effect, under the hypothesis of efficacy, is above 1.96 SE’s. For the effect to be confidently above this level (e.g. 85% chance) its mean should be about 1 SE greater than this (adding 1.96 + 1 gives 3). If the sample sizes in the two groups are n and kn then D will have a standard error of √(V/n + V/kn) = √(V(k + 1)/n), where V is the variance or standard deviation (SD) squared. Note that it has been assumed the SD is the same in both groups, a single applicable standard deviation is required for analysis of variance to be valid. The sampling distributions have been based on the Student’s t distribution rather than the standard normal distribution to allow for greater variation with smaller sample sizes. Transformation to normality with a log transformation has also been mentioned, this is relevant if the data are positively skewed and variability is better summarised by the coefficient of variation (CV) rather than the SD of the untransformed values. In the calculations employed the SD is corrected for the number of samples/measures employed using the variance inflation factor. For binomial sample sizes the estimated variance of D is based on the weighted average of the two rates under H_0_ and H_1_, the weighting being according to the proportion of subjects in each group. Formulae for CI for ICC [14, 15], ICC Sample size [16], Cronbach’s alpha [17] are also given in the relevant sections of STARS. The formulae used in Excel can be seen for all calculations on the relevant worksheets, these have to be unprotected to view them, the password is given on the Contents worksheet.

The model that is assumed for continuous normal variables is:

$${\mathrm{y}}_{\mathrm{i}\mathrm{j}}=\mathrm{c}+{\upbeta}_{\mathrm{i}}+{\mathrm{u}}_{\mathrm{i}}+{\upvarepsilon}_{\mathrm{i}\mathrm{j}}$$

where

y_ij_ is the jth measure in the ith subject,

c is the intercept (a constant representing the baseline effect, e.g. in the reference group).

β_i_ is the between-group effect to be detected, it is zero for the reference group and is the difference to be detected for the second group.

u_i_ is the random between-subject variation, it is normally distributed with a mean of zero.

ε_ij_ is the random within-subject variation, it is also normally distributed with a mean of zero.

This model cannot be used for paired measurements such as might be employed for estimation of the difference between pre and post-treatment effects, these would typically be based on the paired t-test, or McNemar’s test in the case of binomially distributed outcomes.

## Results

### The relationship between sample size and ICC

**Fig. 2:**
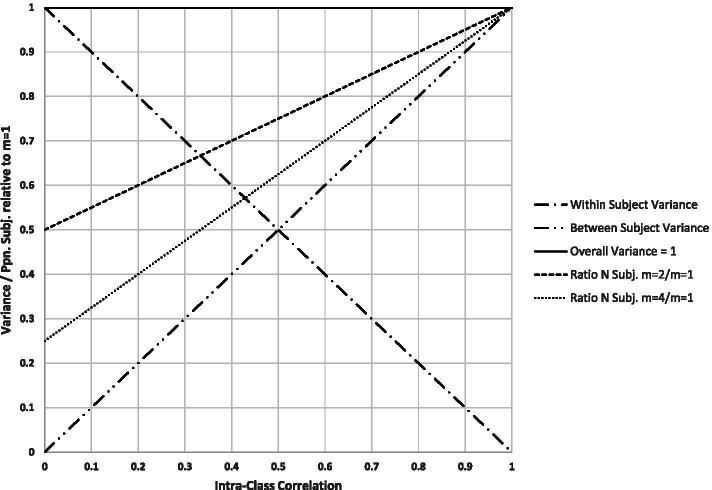
A schematic illustrating the relationship of overall variability, between-subject variability, within-subject variability, intra-class correlation (ICC), number of synchronous samples (m), and study size. For convenience, overall variability is set to 1, between-subject variability is the increasing diagonal (left to right), and within-subject variability the decreasing diagonal. The intra-class correlation (ICC) is on the x-axis and summarises the relationship between subject and within-subject variability. Study size is statistically linearly related to the two sources of variability and correlation and in addition to the number of synchronous samples (m)

The measurement variance is composed of between-subject variability and within-subject variability (Fig. 1A). Hypothetically, if the overall variance is considered constant and the within-subject variance decreases, then the between-subject variance must increase proportionately (Fig. 2). The ICC (x-axis, Fig. 2) is where the specific between/within variance relationship occurs for the measure. The ICC is the proportion of the overall variation attributable to between-subject differences, calculated as the between-subject variance divided by the overall variance (set to 1 here). The relationship between ICC and the % division of the two sources of variance is the centered ‘X’ in the plot.

The sample size chosen for a clinical trial or other between-group study comparison is directly related to the variation of the outcome. For example, setting out to detect a 1 unit increase between two equal-sized groups and considering outcome variances of 1, 2, and 4, appropriate calculated sample sizes might be 146, 292, and 584 subjects, respectively; these also have a 1:2:4 ratio. In Fig. 2, the relative numbers of study subjects required for different ICCs with m = 2 and m = 4 are shown as the upper diagonal line starting with 50 and 25% of subjects, respectively. The pattern shown in Fig. 2 is also shown in Table 1, with alternative designs for specific ICCs being shown in columns.

**Table 1:** The relationship between the proportion of measures and subjects versus intra-class correlation relative to the proportion needed when only one measure is employed (top rows). The lower table shows the effective percentage increase in study efficiency (in terms of subjects) that can be achieved by taking multiple samples. For example, a two-sample study with an ICC of 0.5 is equivalent to a one-sample study with 33% more subjects (as is also apparent from the first table in the 75 to 100 difference)

%Measures/% Subjects								
**Intra-Class Correlation**								
**Measures**	0	0.15	0.35	0.5	0.65	0.85	0.9	1
1	100 / 100	100 / 100	100 / 100	100 / 100	100 / 100	100 / 100	100 / 100	100 / 100
2	100 / 50	116 / 58	136 / 68	150 / 75	166 / 83	186 / 93	190 / 95	200 / 100
3	99 / 33	129 / 43	171 / 57	201 / 67	231 / 77	270 / 90	279 / 93	300 / 100
4	100 / 25	144 / 36	204 / 51	252 / 63	296 / 74	356 / 89	372 / 93	400 / 100
5	100 / 20	160 / 32	240 / 48	300 / 60	360 / 72	440 / 88	460 / 92	500 / 100
**Effective Sample Size**								
**Measures**								
1	100	100	100	100	100	100	100	100
2	200	174	148	133	121	108	105	100
3	300	231	176	150	130	111	107	100
4	400	276	195	160	136	113	108	100
5	500	313	208	167	139	114	109	100

For an ICC of 0.5, 100 samples could be taken from 100 patients (with m = 1), or 201 samples could be taken from 67 subjects (with m = 3). If the recommended study size with one sample were 160 patients, the corresponding figures for m = 3 would be 1.6 times the values shown (324 samples in 108 patients). Some scenarios are unlikely to ever be of practical value, such as those with ICCs of 0.85 or greater because of the small efficiency improvement. A similar table, with 5% ICC increments, is given in the Excel workbook (STARS.xlsx, ‘Introduction’ worksheet), together with an example of data analysis, with associated R code (STARS.xlsx, ‘Analysis Examples’ worksheet).

For binary endpoints the considerations are similar. For example, if two measures are being considered and there is a high correlation within subjects, then the response to the first measure will indicate what the response to the second measure is likely to be. Researchers’ decisions will vary according to context and simulation may be valuable in this context. A two-measure example is presented in the software (see ‘Binomial ICC’ worksheet for link).

The Excel workbook (STARS.xlsx), which accompanies this paper, contains worksheets that allow calculation of sample sizes for continuous normal and binomial outcomes, as well as for estimating intraclass correlation. These worksheets can be accessed via the initial ‘Contents’ worksheet. STARS stands for ‘Sample size calculations for Two-group comparisons with Repeated Synchronous sampling.’ All formulae employed can be found on associated ‘Calculations’ worksheets; this format has the advantage of transparency and ease of further development by interested researchers. The worksheets are protected to prevent inappropriate changes, but can be unprotected by using the supplied password. Four features apparent from the above comments and use of this program will now be discussed.

### Increasing power and the available alpha

**Fig. 3:**
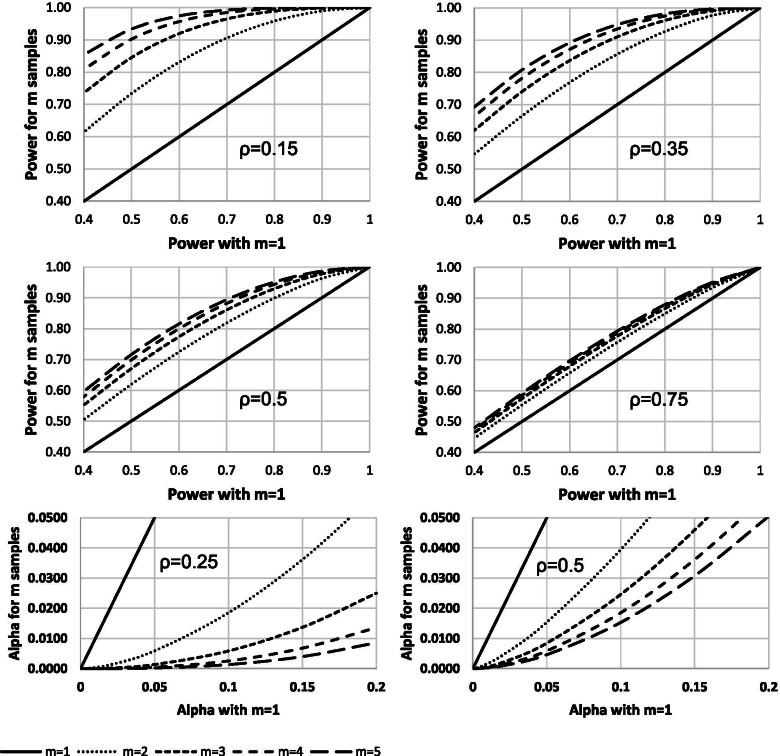
Improvements in power and alpha that can be achieved by increasing the number of measurements and their relationship to the correlation. For power (top four panels), a two-sided significance level of 5% has been used (and a CV of zero, see below). Critical alpha values (bottom two panels, power 85%) can be lowered by making multiple measurements (see text for explanation). The programs for generating these relationships are available in the Excel Web appendix (STARS.xls, under ‘Other’)

If the number of subjects available for a study is approximately known, then because the standard error of the endpoint is reduced by taking multiple samples, increasing the number of measures can be used to increase the power of the study or increase the amount of alpha available. The first four panels of Fig. 3 show power improvements possible for four different ICCs. The final two panels illustrate changes in available alpha. Subjects (such as patients) might be willing to donate more samples or provide more assessments in a study if a benefit was that there were more interim futility or efficacy analyses, or greater power.

As a simple example, if it is decided approximately 200 subjects could be entered into a trial with a single measurement that used an alpha error rate of *P* = 5% for the primary comparison, taking two measurements with an ICC of ρ = 0.5 would imply *P* = 1.54% could be used for this comparison. The remaining 3.46% could be used for other purposes such as interim analyses, and the overall type I error rate of 5% would be maintained.

### Unequal number of samples per subject

There may be circumstances in which there is an unequal number of samples per subject, for example, for logistic reasons or because of subject preference. If the variation in the number of samples is small (CV not greater than 0.23 [18]) the average number of samples per subject can be employed for sample size calculations; in other circumstances, an adjustment should be used. The variation can be summarised by its coefficient of variation (the CV is the SD of m divided by mean), and a correction based on this can be employed (Rutherford, Copas, and Eldridge [18]). If the CV of the number of samples is not known at the outset of a study, the study size could be adapted to allow for the observed variation, estimated from an early analysis.

### Summary endpoints for serial measurements

A linear trend across time is an example of a single measure that can be used to summarise serial within-subject measurements. As mentioned above, linear trends can be estimated more accurately by clustering measurements at the extreme range of independent factors but more complex relationships may also exist. Matthews et al. [19] provides an informative introduction to summary endpoints. Simulation can be used to compare the accuracy of estimated effects using strategies to summarise multiple synchronous measurements. Simulation was used to mimic follow-up over 2 years to identify subjects with rapid visual field progression (− 2 dB/year) by Crabb and Garway-Heath [20]; these showed measurement either 2 or 3 times was superior to every 6 months or every 4 months. The ‘Latanoprost for open-angle glaucoma (UKGTS)’ trial [21] accordingly incorporated this approach.

The methodology described in this paper can also be used where complex relationships exist between repeated measures, and it is possible to combine them into one summary endpoint with an SD it is possible to calculate. In some situations, it may be possible to approximate the number of subjects required if a single overall outcome calculated from true serial measurements is being considered, in which each component measure is weighted equally. It should also be realistic to assume that the relationship between the measurements can be characterized by an overall representative single correlation. This approach could be employed to approximate sample sizes for studies based on more complex correlation matrices, these sample sizes could be refined using simulation employing programs such as SUPERPOWER [22].

### Optimising study design on the basis of ‘cost’

The number of subjects varies with the number of measures employed, so if a cost is assigned to each, then the overall cost can be compared. For example, if the cost per subject is 60 and the cost per sample is 10 (and using a specific design^2^), the costs for up to eight measures are: **1** - 12,180; **2** - 9120; **3** – 8640; **4** – 8400; **5** – 8580; **6** – 9000; **7** – 9360 and **8** – 9660. This suggests the optimum number of measures is 4, but the cost for 3 is similar. Cost units could be arbitrary; for example, the assigned cost could represent a currency or an alternative such as a linear score combining cost to the subject in terms of inconvenience and risk, the cost to the staff undertaking the procedure, and financial cost. This result is similar to that obtained from the formula suggested for the optimum number of patients in the clusters of cluster randomized trials (m = √(c/s x (1- ρ)/ρ) [23], which yields m = 3.74, where c is a cost per subject and s the cost per measure. This aspect of study design is also included in the Excel Workbook (STARS.xlsx, Sample Size calculation worksheets).

**Table:** 

**Example**	
A meta-analysis of trials examining blood pressure lowering treatments found that the average (systolic) blood pressure reduction beyond 12 months in placebo-controlled trials was 5.1 mmHg (SE 0.06, *N* = 112,934) [2]. A 5 mmHg reduction was associated with a 10% reduction in the rate of major cardiovascular events, irrespective of cardiovascular disease status at randomization [1].	
Suppose a randomized placebo-controlled phase II trial of a new anti-hypertensive drug was planned, with the intention of detecting a 5 mmHg fall in systolic blood pressure at 12 months, preparatory to a larger phase III trial with cardiovascular events as an endpoint. The SD of the fall is assumed to be 12.5 mmHg and the cost of enrolling a subject is estimated to be 15 times the cost of making an additional measurement on an enrolled subject. The ICC for repeat systolic measurements is assumed to be 0.67 [3]. The use of multiple measures at baseline and 12 m is being considered. STARS suggests the use of 178 subjects (89 per group, 85% power, two-sided 5% significance level), with three measures at these timepoints per subject or 190 subjects with two measures. The cost of three or two measures is 12% or 11% less than employing one measure, respectively, which would require 228 subjects.	
References	
1) 10.1016/S0140-6736(21)00590-0	
2) https://www.medrxiv.org/content/10.1101/2021.02.19.21252066v1	
3) Morgan et al. [5]	

## Discussion

If there is an opportunity to repeat assessments in a study, it is useful to quantify the benefit of this strategy. There is a danger that the use of multiple assessments is dismissed too readily, perhaps merely on the basis that assessments will be correlated, without thoroughly evaluating the value of adding further samples or measures. The additional burden of extra samples needs to be considered. If a medical study is being prospectively defined, which requires assessments over and above those of standard care, public/participant involvement (PPI) could be employed to investigate participants’ views on the provision of extra samples balanced against the benefits this could yield in design. These could include a smaller overall study size, shorter trial duration, increased power, or more interim analyses. In studies using laboratory animals, smaller experiments may be desirable to reduce the number of animals required, particularly if they have to be sacrificed. Note also that if an outcome is of interest, high within-subject variability does not necessarily preclude a study from being undertaken if it is possible to take multiple samples. A small, intensive study of the value of an outcome with high within-subject variability may sometimes be useful to evaluate whether it is worth refining measurement of the outcome to reduce within-subject variability. The cost of some samples or measures may be reduced with time as more efficient methods of obtaining and analyzing them are developed, making it more practical to obtain multiple samples. It may therefore be useful to review decisions when sample costs decrease.

It is important to note that the scale of measurement can be critical when measuring ICCs. Repeatability is frequently assessed by plotting the values of two measurements on the same subject against each other as a scatterplot with a line of equality. However, the variability is more easily understood by plotting the difference in a subject’s measurements from the two methods against the mean of the measurements, known as a Bland–Altman plot [24]. These plots illustrate measurement error alongside the necessary ‘limits of agreement’, which give a range within which 95% of future differences in measurements would be expected to lie, the latter being calculated from the mean and SD of the paired differences [25]. However, this method assumes the SD is the same throughout the measurement range. It is common for the SD to increase with the mean in biological contexts, the coefficient of variation (CV) rather than the standard deviation often being quoted to summarise variability for such measurements. This suggests the measurements have a lognormal rather than a normal distribution and a remedy that is frequently successful is to use the logarithm of the two measurements for analysis [25].

A further consideration is that studies that focus on subgroups for precision medicine may have a lower ICC than an unselected group of subjects, making repeated assessments more relevant. If a prognostic factor is used to select a subgroup with a more limited outcome range, then the between-subject variance of this range would be expected to be lower than that in all subjects. However, the within-subject variance might be expected to be similar, lowering the ICC. When focussing on subgroups multiple measurements per subject may therefore be relevant, but careful consideration of the context is needed.

Given the potentially high variability seen in high throughput assays referred to in the introduction, it is interesting to note that individual results of components of high throughput assays are sometimes aggregated to produce a single overall score to represent an underlying phenomenon of interest. This may offer a way to improve study efficiency without making more measurements because the averaging across several components could even out the effect of errors in the individual components. For example, a multigene assay score that is used to predict recurrence in Breast Cancer [26] has a proliferation (tumor growth) component that is composed of the expression of five genes (Ki67, STK15, Survivin, CCNB1, and MYBL2) combined by averaging the five gene scores. It would be expected that the components will be correlated if such an approach is used.

The need for a representative sample may override the desire to reduce the number of subjects by making multiple measurements. The majority of studies aim to obtain a typical sample of the population of subjects being examined to ensure the study results are generalizable. For example, a study of 40 subjects might not be considered large enough to represent the diversity seen in the population the study was chosen to represent. However, a study of 100 subjects may be considered more appropriate.

Note that systematic differences between repeats do not necessarily invalidate the use of repeat samples. It may be possible to adjust the repeat measurements for prognostic factors to quantitatively remove such differences; this may have the effect of increasing the ICC.

## Conclusion

It may be beneficial to undertake multiple synchronous observations per subject in some circumstances. This option is part of the toolset available to researchers when planning effective studies. Both between-subject and within-subject variability are critical parameters for decision-making in this context. An Excel workbook is provided to aid exploration of the statistical background of this feature of study design.

## Availability and requirements

Project name: STARS

Project home page: None

Operating system(s): Windows with Microsoft Office and Excel

Programming language: Only formulae available in Excel are employed

Other requirements: None

License: None required

Any restrictions to use by non-academics: None

## Supplementary Information

**Additional file 1.** STARS.xlsx

## Data Availability

One item, an Excel Workbook, submitted. It contains previously published anonymous data.

## Abbreviations

CV: Coefficient of variation
ICC: Intra-class correlation coefficient
STARS: Sample size calculations for Two-group comparisons with Repeated Synchronous sampling

## Footnotes

1 If **D** = (− 1, 1) and **X** = (pre, post) then **DX**^T^ is the post-pre score for each subject, where **X** represents the data and **X**^T^ is **X** transposed.

2 Difference to detect = 0.5, SD = 1, ρ = 0.3, CV = 0.5, Power = 0.85, alpha = 0.05, k = 2.
